# Effect of fertilization combination on cucumber quality and soil microbial community

**DOI:** 10.3389/fmicb.2023.1122278

**Published:** 2023-02-23

**Authors:** Mei Wang, Yu Xu, Haiping Ni, Shiai Ren, Ni Li, Yuxia Wu, Yan Yang, Yumin Liu, Zongzheng Liu, Yingchun Liu, Jing Shi, Youming Zhang, Lihua Jiang, Qiang Tu

**Affiliations:** ^1^Institute of Agricultural Resources and Environment, Shandong Academy of Agricultural Sciences, Jinan, China; ^2^Helmholtz International Lab for Anti-Infectives, State Key Laboratory of Microbial Technology, Shandong University–Helmholtz Institute of Biotechnology, Shandong University, Qingdao, China; ^3^CAS Key Laboratory of Quantitative Engineering Biology, Shenzhen Institute of Synthetic Biology, Shenzhen Institute of Advanced Technology, Chinese Academy of Sciences, Shenzhen, China; ^4^Qingdao Institute of Animal Husbandry and Veterinary Medicine, Qingdao, China; ^5^College of Resources and Environmental Engineering, Shandong University of Agricultural Engineering, Jinan, China

**Keywords:** biomarker microorganisms, bacterial community, fertilizer, fungal community, manure, soil organic matter

## Abstract

Due to the lack of scientific guidance on the usage of fertilizer, the overuse of chemical and organic fertilizer is commonly witnessed all over the world, which causes soil degradation and leads to environmental pollution. The effect of fertilizer strategies on soil properties, cucumber nutrients, and microbial community was investigated in this study with the aim to explore an optimized and enhanced fertilizer strategy. There were five fertilizer strategies conducted including CK (no fertilizer), M (cow dung manure only), NPK (chemical fertilizer only), NPKM (chemical fertilizer combined with manure), and DNPKM (30%-reducing chemical fertilizer combined with manure). It was found that different fertilizer strategies significantly affected the soil organic matter and nutrient levels and cucumber production and nutrient contents of the experimental field. Different fertilizer strategies showed dramatic effects on the alpha- and beta-diversity of soil microbial communities. Moreover, NPKM and DNPKM groups could significantly improve the bacterial abundance and fungal diversity. In addition, the structure of microbial communities was significantly changed in the presence of manure, chemical fertilizer, and their combination. Optimized combination of NPK with M improved the abundance of aerobic, biofilm formation-related, and Gram-negative bacteria and suppressed the anaerobic and Gram-positive bacteria. The presence of saprotrophs fungi was enhanced by all fertilizer strategies, especially the plethora of *Gymnoascus*. The combination of manure with chemical fertilizer could improve the availability of nutrients, and therefore reduce the adverse effects and potential risks induced by excessive fertilizer application. In conclusion, the new fertilization approach can not only meet the growth requirements of cucumber after reduced fertilization, but also protect soil health, which provides a new candidate for the eco-friendly technology to satisfy the topic of carbon neutrality.

## Introduction

Greenhouse vegetable production is a kind of intensive production with high investment and high output, which is an important component of the vegetable growing system in China (Zheng, 2000). According to the latest statistics, the vegetable growing area of China accounts for about 42% of the total vegetable sown area worldwide (Boliko, 2019). Cucumber is one of the most popular vegetables in greenhouse vegetable production, and the planting area and production of cucumber are increasing gradually. It was reported that the planting area of cucumber in China accounted for about 56% of the total growing area of cucumber around the world and its production accounted for over 80% of the worldwide production of cucumber (Feng et al., 2020).

Heavy chemical fertilizers and light organic fertilizers have been widely applied in vege production, especially greenhouse vegetable production (Liu et al., 2014). The average fertilizer nutrient in the greenhouse vegetable production vividly exceeds the used number of other crops, which leads to soil nutrients overaccumulation, decreased soil organic matter, and destroyed soil microbial diversity, which seriously impaired the sustainable development of greenhouse vegetable production (Yang et al., 2016; Pu et al., 2020; Wei et al., 2020; Zhao et al., 2021). Due to the overuse of chemical fertilizers and farmyard manure, a variety of planting impediments have been found, such as soil acidification, salinization, and nutrient imbalance (Huang et al., 2011; Ti et al., 2015). The lack of scientific guidance on the use of organic fertilizers is another important factor in the wide variation in the effectiveness of fertilizers.

The rhizosphere is a microenvironment that is related to the interaction between roots and soil microorganisms and consequently influences plant growth (Qu et al., 2020). Soil microorganisms are critical members of the farmland ecosystem, which could also indicate the quality of soil health and fertility (Lugtenberg and Kamilova, 2009; Emmett et al., 2020). The structure and diversity of soil microbial communities are sensitive to external environmental factors, such as fertilization and irrigation strategies (Bhattacharyya et al., 2017; Chen et al., 2020; Dangi et al., 2020; Khan et al., 2021). For example, Dang et al. (2019) found that long-term wastewater irrigation dramatically affected the alpha and beta diversity of the soil microbial community, which was found to be greater than the effect of soil depth. A former study reported that the application of chemical fertilizers and biochar could significantly affect the fungi community of acidic soils, and soil nutrient-related fungi were found to be differentially expressed, which could serve as potential biomarkers (Zhang et al., 2019). Wu et al. (2021) reported that the combination of mineral and organic fertilizers could improve soil microbial biomass and increase soil macroaggregates in saline-alkaline soil (Wu et al., 2021). Luan et al. replaced chemical fertilizers with manure and found that manure improved soil organic carbon, available nitrogen, and phosphorus and promoted the growth of microorganisms (Luan et al., 2020).

This study aimed to unveil the effect of fertilizer strategies (no fertilizer in the medium-long term, only chemical fertilizers or manure, and combination of chemical fertilizer and manure with different proportions) on soil nutrients and microbial communities. We will support sustainable green agriculture by providing a practical approach for farmers to maximize crop yield and quality without compromising the health of the soil.

## Materials and methods

### Experiment sites and study design

Field experiments were conducted in a greenhouse at Dayu Village (37.11°N, 117.28°E), Qudi Town, Jiyang District, Jinan, Shandong Province, China. Three-years cucumber production was evaluated with two crops cycle a year. The specific time points of each crop were summarized in Supplementary Table S1. The basic physicochemical properties of the experiment sites are summarized in Supplementary Table S2. The autumn and winter stubble variety were Jinyou 208 and the spring stubble variety was Deruit Y2.

There were five treatments with three replicates of each in this study. The plot area was 32 m^2^, and an obvious border was established between plots. The specific fertilizer managements of each group are as follows: (1) CK: no fertilizer; (2) M: 2100 kg/acre manure without chemical fertilizers; (3) NPK: optimized chemical fertilizer (N-P_2_O_5_-K_2_O: 30–15-40 kg/acre) only without manure; (4) NPKM: optimized chemical fertilizer (N-P_2_O_5_-K_2_O: 30–15-40 kg/acre) with 2,100 kg manure; (5) DNPKM: 70% optimized chemical fertilizer (N-P_2_O_5_-K_2_O: 21–10.5-28 kg/acre) with 2,100 kg manure.

The manure was cow dung compost with a moisture content of 28.56% and a nutrient composition of 18.64–14.54-13.85 kg/acre (N-P_2_O_5_-K_2_O). The manure was applied disposable as basal fertilizer. The optimized fertilizer was conducted with 9–9-10 chemical fertilizer (formulated with the 15–15-15 compound fertilizer and Potassium sulfate fertilizer) as the basal fertilizer, and the 21–6-30 chemical fertilizer (formulated with the 15–5-25 compound fertilizer and urea) was applied with water topdressing divided into 6–7 times at the three-leaf stage, the early melon stage, and the melon-filling stage. 2.1 kg, 2.2 kg, and 2.2 kg of urea were added during topdressing. For the 70% optimized chemical fertilizer, the basal fertilizer was applied with the 3.98–5.5-3 chemical fertilizer and the 17–5-25 chemical fertilizer was used as the topdressing.

### Soil and plants analyses

Soil samples were collected at the end of the final crop. Soil pH and EC (soil Electric Conductivity, a numerical measure of the ability of a soil solution to conduct current. Units are expressed in Siemens per meter (S/m), 1S/m = 10,000 US/cm) was evaluated with a glass electrode with a soil-water ratio of 1:5. The bulk density, total porosity, and the content of >0.25 mm aggregates were analyzed by ring knife method, calculation method, and dry screen method (Zhang et al., 2022). Soil organic matter (SOM) was measured with the K_2_Cr_2_O_7_. For the nutrient contents, soil available P and K were extracted with the Melich III method according to previous reports (Wang et al., 2007), and the contents of total nitrogen, NO_3_-N, and NH_4_^+^-N were analyzed with colorimetry. Meanwhile, the contents of N, P, and K and the moisture content of the harvested cucumbers were also analyzed.

### Measurement of vitamin C, nitrate, soluble sugar, and soluble solids

Vitamin C (V_C_) was measured using a titration method with 2,6–dichlorophenol (Qi, 2003). Nitrate was analyzed with salicylic acid nitration (Li, 2000). Soluble sugar content was determined by 3,5 dinitrosalicylic acid colorimetric method (Wang et al., 2007). Soluble solids were analyzed with a fully automatic refractometer (RX-5000a, ATAGO CO., Ltd., Japan) according to Wang et al. (2007)).

### DNA extraction, PCR amplification, and sequence analysis

DNA extraction was conducted with the employment of the EZNA soil DNA kit (Omega Bio-Tek, United States) according to the manufacturer’s instructions. The concentration, quality, and purity of the isolated DNA were assessed by the NanoDrop 2000 (Thermo Fisher Scientific, United States) with 1% agarose gel electrophoresis for the evaluation of DNA quality.

The PCR amplification was performed with the 338F and 806R primers for the bacterial V3-V4 region, and the ITS1F and ITS2R for the fungal ITS hypervariable region. The sequences of used primers are summarized in Supplementary Table S3. The sequence analysis was carried out in accordance with the processes shown in Supplementary Figure S1 by Beijing BMK Biotechnology Co (Beijing, China).

### Data processing

Raw data were merged using FLASH v1.2.11 and filtered by Trimmomatic v0.33 (Adekiya et al., 2020). The chimera sequences were identified and removed using the UCHIME version 8.1, and the high-quality Tags were obtained (Edgar et al., 2011). Clean tags were clustered into OTU by USEARCH (version 10.0) at 97% similarity levels (Edgar, 2013). The OTU was filtered when the readvance was less than 0.005%.

The species annotation and taxonomic analysis were performed based on the Silva database (for bacteria)^1^ and the Unite database (for fungi)^2^ and the threshold was set as 0.8.

### Statistical analysis

The soil and plant data were analyzed by one-way ANOVA followed by the Duncan post-hoc test. Significant differences between the means of treatments were analyzed by a one-way analysis of variance procedure (ANOVA) at a *p* < 0.05 level using DPS software and indicated by different letters. The sequence data were compared using R software. Specifically, the alpha-diversity was evaluated by several major indexes, including ACE, Chao1, Simpson, Shannon, and coverage, which were compared using student’s t-test. The beta-diversity was assessed by the principal coordinate analysis based on Bray-Curtis, Jaccard, weighted-unifrac, and unweight unifrac distance algorithms.

The differential microbial communities and species were identified using Lefse with the LDA threshold 4. Moreover, the function prediction was conducted with the help of PICRUSt2 (for bacteria) and FUNGild (for fungi). The co-expression network was also conducted to estimate the interaction between different genera with Spearman correlation analysis (|*r*| > 0.1, *p* < 0.05).

## Results

### Effect of fertilizer strategies on soil physicochemical properties

Compared with CK, M significantly improved soil total nitrogen, but NPK dramatically increased EC and total nitrogen content and reduced SOM. The combination of manure with NPK showed a remarkable impact on the SOM compared with their single application, and the maximum contents of AP, AK, and TN were also found in this treatment. Interestingly, DNPKM showed a maximum SOM content and lower TN content than other fertilizer strategies (Table 1). Additionally, no significant difference was observed in the content of >0.25 mm aggregates. M significantly improved soil bulk density and porosity, and the treatment of DNPKM showed a significant result (Table 2).

**Table 1:** Soil physicochemical properties of different fertilizer treatments.

	pH	EC (US/cm)	SOM (g/kg)	AP (mg/kg)	AK (mg/kg)	TN (g/kg)
CK	7. 66 ± 0.03	510.67 ± 2.89	36.31 ± 1.13	412.05 ± 17.64	525.00 ± 13.23	2.35 ± 0.03
M	7.64 ± 0.01	551.33 ± 7.09	38.47 ± 1.07	434.17 ± 4.67	535.00 ± 5.00	2.75 ± 0.04^**^
NPK	7.66 ± 0.01	572.33 ± 30.29^**^	32.69 ± 1.07^*##^	411.47 ± 25.89	545.00 ± 22.91	2.69 ± 0.21*
NPKM	7.63 ± 0.08	590.33 ± 13.80^***^	37.78 ± 2.60^*+^	444.76 ± 11.07	568.33 ± 12.58*	2.85 ± 0.11^**^
DNPKM	7.67 ± 0.08	575.33 ± 7.02^**^	39.46 ± 0.83^++^	430.84 ± 3.56	538.33 ± 5.77	2.57 ± 0.05^+^

SOM, soil organic matter; AP, available phosphorus; AK, available potassium; TN, total nitrogen. *p < 0.05 relative to CK; ^#^*p* < 0.05 relative to M; ^+^*p* < 0.05 relative to NPK; ^$^*p* < 0.05 relative to NPKM.

**Table 2:** Soil physical properties of different fertilizer treatments.

	Bulk density (g·cm^−3^)	Total porosity (%)	>0.25 mm aggregate (%)
CK	1.26a ± 0.05	50.93c ± 1.80	36.14a ± 2.00
M	1.18b ± 0.06	54.15b ± 3.09	36.21a ± 1.53
NPK	1.20ab ± 0.03	53.59bc ± 0.85	37.29a ± 1.42
NPKM	1.17b ± 0.02	54.79b ± 0.80	36.58a ± 1.57
DNPKM	1.08c ± 0.04	58.53a ± 1.12	37.47a ± 0.80

Values are the means ± standard error of three replicates. Different lowercase letters indicate a significant difference between the treatments at the *P* < 0.05 level.

### Effect of fertilizer strategies on cucumber production and nutrient contents

Compared with CK, all fertilizer treatments significantly improved the 3-year production of cucumber. Specifically, NPK (688.82 ± 13.99 t/ha) and NPKM (660.09 ± 12.63 t/ha) showed a maximum production followed by DNPKM (656.24 ± 4.78 t/ha, Table 3). The specific production of each crop is summarized in Supplementary Table S4.

**Table 3:** 3-year production and nutrient contents of cucumber in different fertilizer treatments.

	Production (t/ha)	N (kg/ha)	P_2_O_5_ (kg/ha)	K_2_O (kg/ha)
CK	605.68 ± 11.15	65.33 ± 3.07	41.16 ± 1.72	93.55 ± 4.24
M	625.34 ± 6.09*	75.66 ± 1.33	42.04 ± 0.95	96.57 ± 1.78
NPK	688.82 ± 13.99^*#^	106.82 ± 12.12^*#^	70.45 ± 4.13^*#^	135.90 ± 10.78^*#^
NPKM	660.09 ± 12.63^*#^	96.50 ± 13.32*	60.64 ± 12.66^*#^	120.07 ± 15.90
DNPKM	656.24 ± 4.78^*#+^	99.96 ± 14.98*	62.95 ± 7.15^*#^	127.78 ± 12.21^*#^

*p < 0.05 relative to CK; ^#^*p* < 0.05 relative to M; ^+^*p* < 0.05 relative to NPK.

In case of nutrient contents in cucumber, all the fertilizer treatments showed a significant effect on the contents of N and P_2_O_5_ in cucumber compared with CK, and NPK and DNPKM dramatically enhanced the content of K_2_O in cucumber. Compared with M, NPK showed apparent enhancement in the contents of N, P_2_O_5_, and K_2_O in cucumber, while NPKM significantly improved the contents of P_2_O_5_, and DNPKM dramatically increased the contents of both P_2_O_5_ and K_2_O (Table 3).

Moreover, the effects of fertilizer strategies on the content of V_C_, nitrate, soluble sugar, and soluble solids were also evaluated, where soluble solids and nitrates showed a strong response. In the treatment of NPK with the highest yield, the contents of V_C_, soluble sugar, and soluble solids were relatively lower, suggesting that the single application of chemical fertilizer could possibly increase output but reduce quality. M, NPKM, and DNPKM showed a better quality than NPK and CK, where the content of Vc in NPKM and DNPKM increased 18.90 and 8.82%; the content of soluble sugar increased 16.40 and 14.29%; the soluble solids improved 12.23 and 17.12% compared with NPK, respectively, indicating the great improvement of cucumber quality (Table 4). Compared with NPKM, DNPKM reduced the fertilizer consumption by 30% and nitrate content of cucumber by 21.79%, suggesting higher food safety factor.

**Table 4:** The cucumber quality of each crop.

	V_C_ (mg/kg)	Nitrate (mg/kg)	Soluble sugar (%)	Soluble solids (%)
CK	167.53ab ± 8.59	103.66b ± 2.20	2.01bc ± 0.04	3.17c ± 0.06
M	180.02a ± 10.87	133.15a ± 14.34	2.19a ± 0.06	3.50b ± 0.17
NPK	129.94d ± 17.56	134.51a ± 4.21	1.89c ± 0.14	3.27c ± 0.12
NPKM	154.50bc ± 11.71	141.55a ± 5.32	2.20a ± 0.11	3.67ab ± 0.12
DNPKM	141.40 cd ± 10.98	110.70b ± 5.84	2.16ab ± 0.11	3.83a ± 0.15

Values are the means ± standard error of three replicates. Different lowercase letters indicate a significant difference between the treatments at the *p* < 0.05 level.

### Effect of fertilizer strategies on the alpha-diversity of soil microbial community

**Figure 1:**
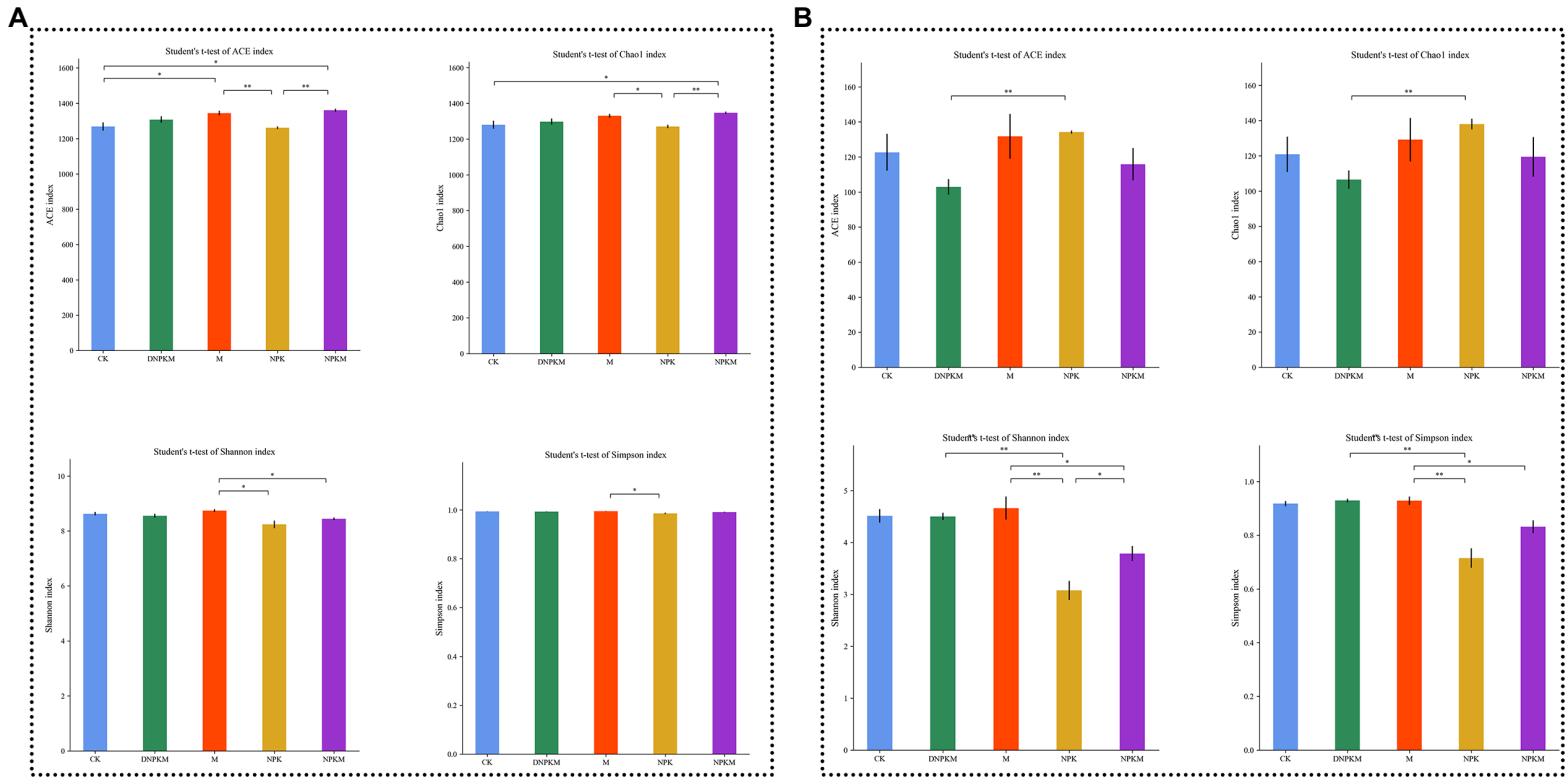
Effect of fertilizer strategies on the alpha-diversity index (ACE, Chao1, Shannon, and Simpson) of soil bacterial community **(A)** and fungal community **(B)**. *p < 0.05, **p < 0.01.

The ACE and Chao1 indexes were used to indicate the abundance of the bacterial community, while the diversity was indicated by the Shannon and Simpson indexes. It was found that M significantly improved the abundance compared with CK. While compared with M, soil with only chemical fertilizer showed a lower opulence and diversity, but NPKM dramatically increased the ACE and Chao1 indexes. No significant changes were observed after reducing chemical fertilizers (DNPKM, Figure 1A).

For the fungal community, no significant difference was observed in the abundance and diversity between fertilizer treatments and CK. But in comparison with M, both NPK and NPKM showed considerably reduced diversity of the fungal community, and a significant difference was found between NPK and NPKM. Interestingly, DNPKM significantly improved fungal diversity compared with NPK (Figure 1B).

### Effect of fertilizer strategies on the beta-diversity of soil microbial community

**Figure 2:**
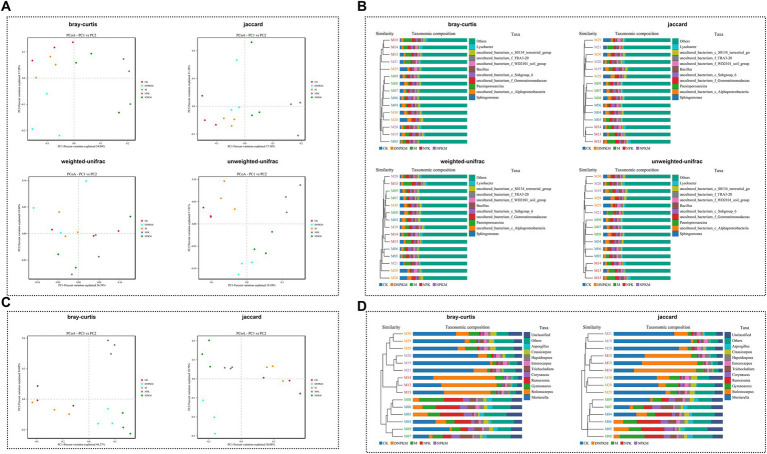
Effect of fertilizer strategies on the structure of soil microbial communities. **(A,B)** PCoA **(A)** and cluster analysis **(B)** of the bacterial community based on the bray-Curtis, Jaccard, weighted-unifrac, and unweighted-unifrac distance algorithm. **(C,D)** PCoA **(A)** and cluster analysis **(B)** of the fungal community based on the bray-Curtis and Jaccard distance algorithm.

All the Bray-Curtis, Jaccard, weighted-unifrac, and unweight unifrac distance algorithms showed a clear separation between different fertilizer strategies (Figure 2A). The hierarchical clustering tree further revealed that the NPK, NPKM, and DNPKM significantly changed the structure of the bacterial community, while combination treatments (NPKM and DNPKM) were substantially different from NPK (Figure 2B).

The NPK, NPKM, and DNPKM were separated from CK and M, and these three groups also showed clear separations from each other (Figure 2C). Consistently, the hierarchical clustering tree showed that the fungal community was significantly affected by the application of NPK, NPKM, and DNPKM, and the fungal structure was completely different between the treatment groups (Figure 2D).

### Identification of differential microorganisms between different fertilizer strategies

**Figure 3:**
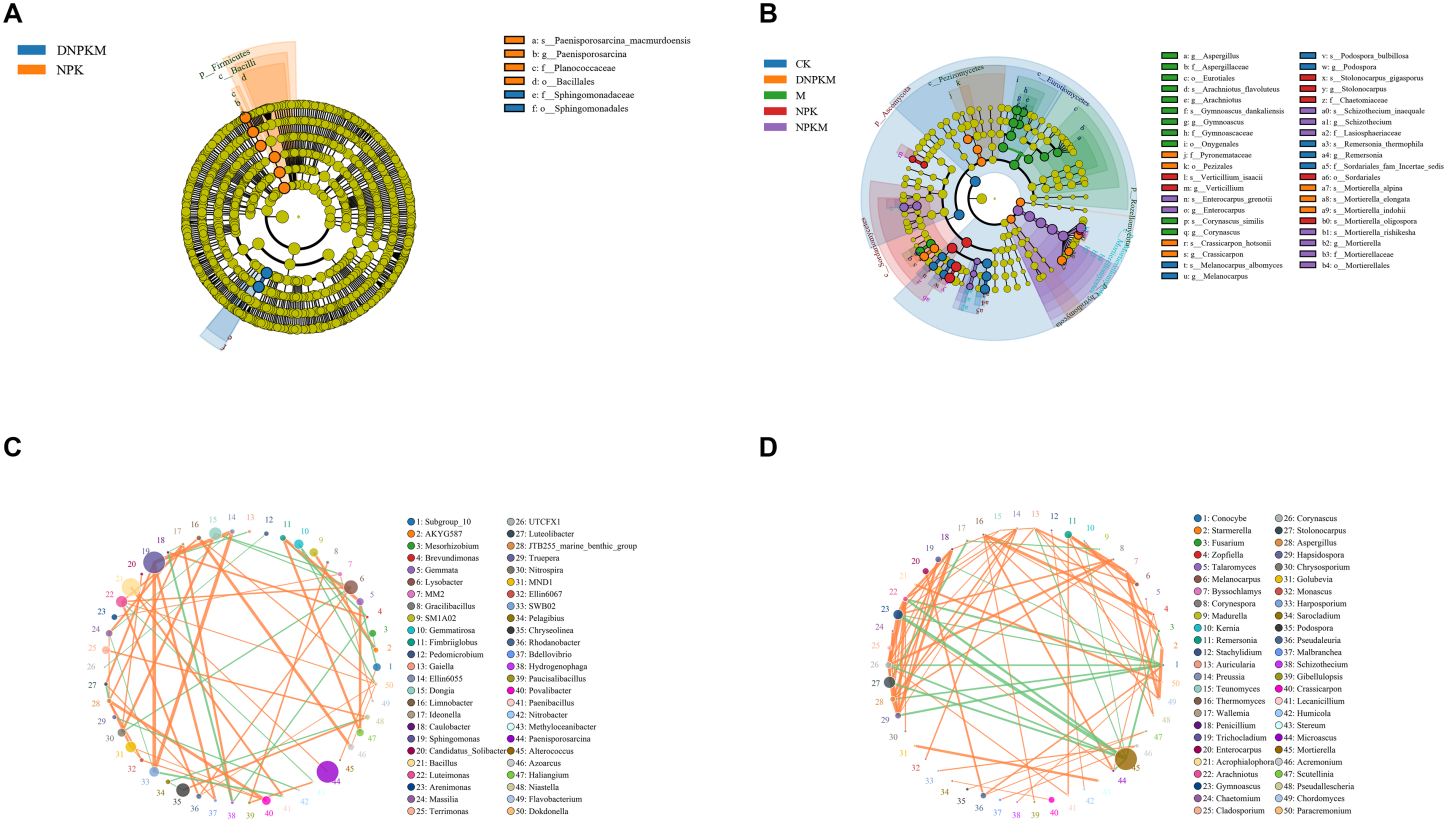
Lefse analysis to identify differential bacteria **(A)** and fungi **(B)** among different treatments. o, order; f, family; g, genus; s, species. The co-expression network of the bacterial community **(C)** and fungal community **(D)**.

A total of 6 differential bacteria and 49 differential fungi were exposed between different fertilizer treatments. The differential bacteria were mainly identified in the NPK (4 biomarkers) and DPKM (2 biomarkers) treatments, including the *Bacillales* order, *Firmicutes* phylum, *Bacilli* class, *Planococcaceae* family, *Paneisporosarcina* genus, and the *Paenisporosarcina macmurdoensis* specie in NPK, and the *Sphingomonadaceae* family and the *Sphingomonadales* order in DNPKM (Figure 3A). The abundance of these biomarker bacteria in each treatment is summarized in Supplementary Figure S2.

There were 4 fungal biomarkers identified for the CK group, 11 biomarkers for M, 7 fungal biomarkers for NPK, 9 for the NPKM group, and 7 for the DNPKM. The differential fungal genera enriched in M were identified as *Gymnoascus*, *Corynascus*, *Arachniotus*, and *Aspergillus*, and the enriched fungal genera in NPK were *Stolonocarpus* and *Verticillium*. While in the combination of M with NPK, the differential fungal genus changed to *Mortierella*, *Schizothecium*, and *Enterocarpus*. After reducing 30% of NPK in the combined fertilizer strategies, the *Crassicarpon* was identified as the potential biomarker genera of DNPKM treatment (Figure 3B). The abundance of these biomarker fungi in each treatment is summarized in Supplementary Figure S3.

The co-expression network was established to estimate the association between the bacterial fungi genus. In the bacterial network, *Sphingomonas*, *Bacillus*, and *Paenisporosarcina* genera showed higher abundance, and *Sphingomonas* genera were found to co-expressed with a variety of bacterial genera (Figure 3C). For the fungal network, *Mortierella* was identified to be the most abundant genera with a negative correlation with several genera. It is noteworthy to mention that there were close correlations were observed among *Penicillium*, *Trichocladium*, *Enterocarpus*, *Acrophialophora*, *Arachniotus*, *Gymnoascus*, *Chaetomium*, *Cladosporium*, *Corynascus*, *Stolonocarpus*, *Aspergillus*, and *Hapsidospora* genus (Figure 3D).

### Effect of fertilizer strategies on the function of soil microorganisms

**Figure 4:**
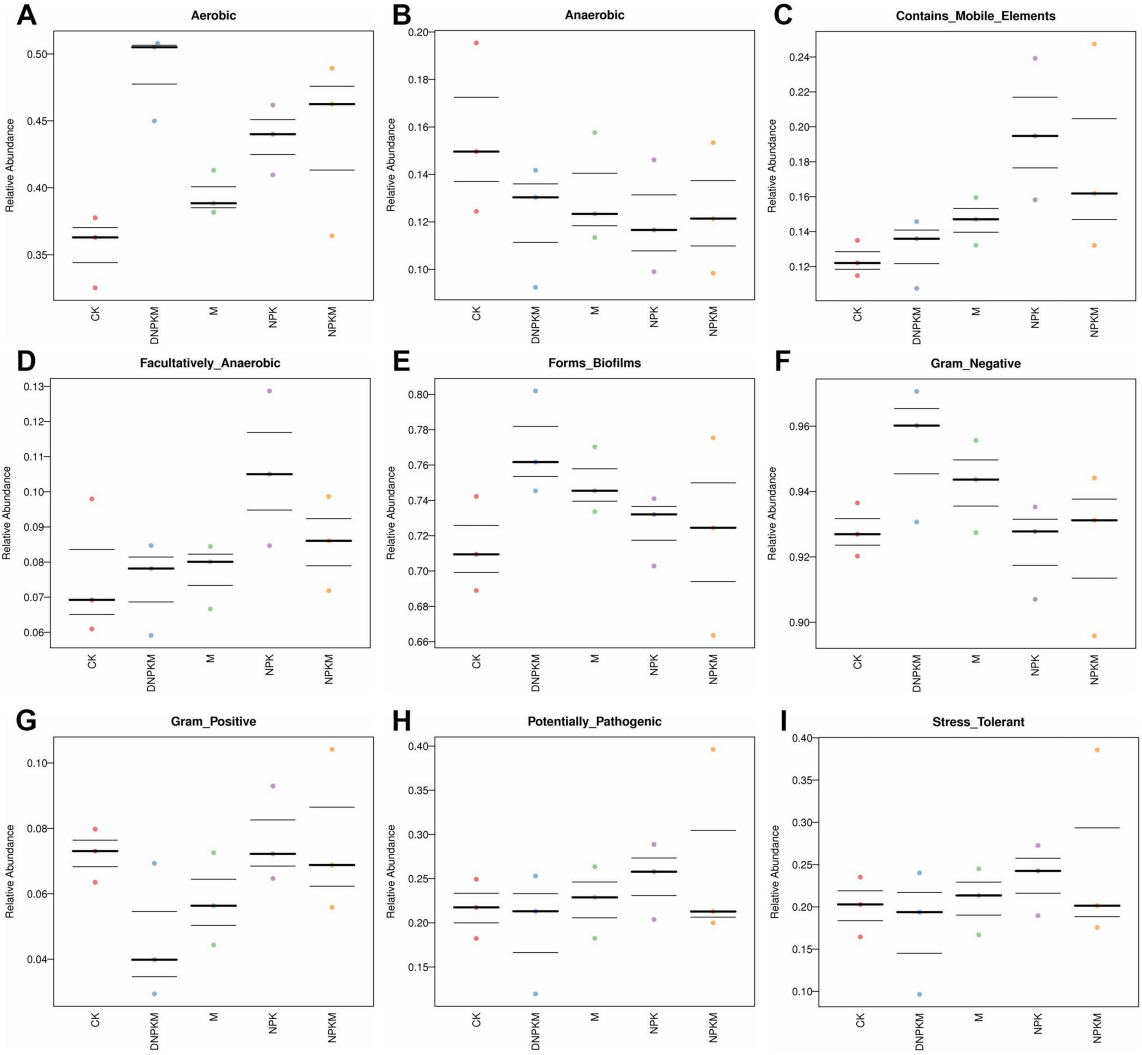
Chart analysis through BugBase software, the head is the function prediction of bacteria.

M (cow dung manure only) improved the abundance of aerobic, biofilm formation-related, gram-negative, mobile elements-containing, and facultatively anaerobic bacteria and reduced the abundance of anaerobic, gram-positive bacteria. While NPK was found to enhance aerobic, mobile elements-containing, facultatively anaerobic, biofilm formation-related and stress-tolerant bacteria abundance, compared with CK. NPKM increased the abundance of aerobic bacteria and reduced the abundance of mobile elements-containing, facultatively anaerobic, biofilm formation-related, gram-positive and stress-tolerant bacteria, while the promoted effect of NPKM on aerobic and inhibitory effect on mobile elements-containing bacteria was enhanced by the reduction of NPK (DNPKM), and the inhibitory effect on potential pathogenic and stress-tolerant bacteria was alleviated by the 30%-reduction of NPK in the combined fertilizer strategies (DNPKM). Moreover, the abundance of gram-positive bacteria was found to be significantly repressed by DNPKM (Figures 4A–I).

**Figure 5:**
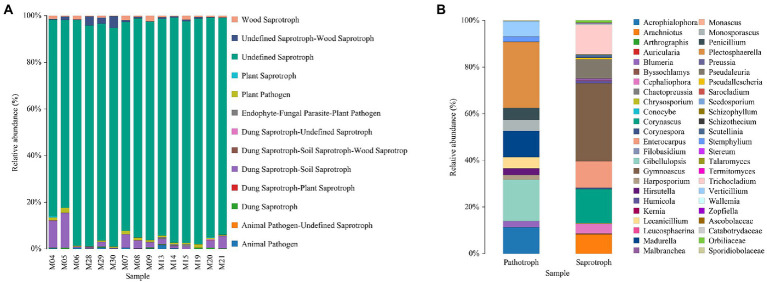
Fungal types in different treatments **(A)** and the distribution of specific genus **(B)**.

For the fungal community, saprotrophs were identified as the most abundant group in each treatment (Figure 5A). The specific abundant genus of the saprotrophs and pathotroph groups are summarized in Figure 5B.

## Discussion

### Effect of fertilizer strategies on soil properties and cucumber growth

Manure and chemical fertilizer are commonly used in agricultural management to improve soil fertility and crop production. It was found that the manure and NPKM in the present study dramatically improved SOM and nutrients level, respectively, whereas NPK without manure was found to decrease SOM. Recently, an increasing number of studies have been devoted to developing optimized fertilizer strategies to improve the utilization of fertilizers and avoid soil degradation (Geng et al., 2019; Iqbal et al., 2020; Ren et al., 2021; Shi et al., 2021). It was investigated in a corn-planting field, the replacement of chemical fertilizer with organic manure improves the activity of soil urease, invertase, and alkaline phosphatase and soil nutrients, which further affected soil physical and chemical properties, including pH, bulk density, and porosity (Ren et al., 2021). Iqbal et al. reported that the combination of chemical fertilizer with poultry or cattle manure could enhance soil health and elevate rice productivity, and the combination of 30% N from manure with 70% N from chemical fertilizer was suggested to be a promising alternative for soil improvement (Iqbal et al., 2020).

From the perspective of cucumber yield, although the yield of NPK was the highest, the difference of NPKM and DNPKM was insignificant. However, in NPKM and DNPKM, the soluble sugar, soluble solid and Vc of cucumber were increased and the nitrate was decreased, indicating the much higher quality of cucumber than NPK. Compared with NPKM, DNPKM reduced the fertilizer consumption by 30% and nitrate content of cucumber by 21.79%, suggesting higher food safety factor. From the perspective of soil health, NPKM groups improved the content of soil organic matter, which is an important indicator of soil health. From the perspective of soil microbial communities, optimized combination of NPK with M improved the abundance of aerobic, biofilm formation-related, and Gram-negative bacteria and suppressed the anaerobic and Gram-positive bacteria. The abundance of Saprotroph fungi was enhanced by all fertilizer strategies, especially the abundance of *Gymnoascus* (Alsabri et al., 2020). Hence, combining these factors, it is recommended that DNPKM might be an optimal choice for most farmers.

In addition, there was no significant difference was observed in the effects of NPK and M, but their combination was found to improve soil EC and SOM, and significantly promoted cucumber production and the nutrients in cucumbers compared with single M and NPK. Interestingly, in the presence of manure, the reduction of NPK did not show adverse effects on soil properties and cucumber production and nutrients, indicating that the combination of manure could counterbalance the effect of NPK and thereby help to reduce the dose of NPK, which could significantly bring down the potential adverse effect of excessive NPK usage. This might be an optimized combination of manure and chemical fertilizer for the growth and yield of cucumber.

### Effect of fertilizer strategies on soil microbial community diversity

Different kinds of manure were shown to exert various effects on soil microbial communities. Green manure could change the structure of the soil fungal community and its effect on plant growth was found to depend on the ratio of carbon and nitrogen (Asghar and Kataoka, 2021). Adekiya et al. (2020) investigated the effect of five kinds of manures, including rabbit manure, cow dung, poultry manure, green manure, and pig manure, on the yield and quality of okra and found that the promoted effect of poultry manure was remarkably higher due to their lower carbon: nitrogen and the lignin: nitrogen ratio.

The cow dung manure was applied in the present study, and it was found that the application of manure could accelerate the bacterial abundance but no significant change was observed in the fungal community. Although the effect of NPK on microbial abundance and diversity was insignificant compared with CK, a significant decrease was found in microbial diversity and bacterial abundance when to compared with M. The bacterial abundance and diversity were enhanced, but the fungal community abundance and diversity was suppressed by the combination of M with NPK. In the presence of manure, reducing 30% of NPK did not affect the alpha-diversity of the bacterial community, but it reduced fungal abundance and improved the diversity. The NPK treatment was carried out according to the usual NPK dosage followed by local farmers, which might be excessive and therefore exerted an inhibitory effect on the alpha-diversity of the microbial community. The reduction of NPK could alleviate the stress of fungi and therefore improve their diversity. The reduced fungal abundance indicated that the reduction of NPK might promote the transformation between some fungal species. The insignificant effect on bacterial community alpha-diversity might result from the relatively lower sensitivity and higher resistance of bacteria to environmental stresses. Moreover, fertilizer strategies strongly influenced the beta-diversity of bacterial and fungal communities, where different treatments were clearly separated from each other and were clustered independently. It was worthy to note that the microbial community between NPKM and DNPKM was not completely separated, suggesting that the reduction of chemical fertilizer did not completely change the microbial community structure (Shi et al., 2019; Jin et al., 2022).

### Effect of fertilizer strategies on microbial community composition and function

The effects of manure and chemical fertilizer on microbial community composition have also been revealed in previous studies and leaked out a series of potential microbial biomarkers. Tang et al. investigated the microbial structure of a double-cropping paddy field and found that manure and chemical nitrogen addition could promote the abundance of phylum *Actinobacteria*, *proteobacteria*, and *Gammaproteobacteria* and fungal phylum *Basidiomycota* and *Zygomycota* (Tang et al., 2020).

Herein, 6 potential bacteria biomarkers and 49 potential fungal biomarkers were identified among different fertilizer strategies. The bacterial biomarkers include 2 orders, 2 families, 1 genus, and 1 species, which is much less than the number of fungal biomarkers, including 5 orders, 7 families, 13 genera, and 24 species, validating the higher sensitivity of fungal community to the fertilizer strategies.

*Sphingomonas* genus was found to possess a high abundance in the bacterial community and show close associations with numerous bacterial genera, such as *Ellin6055* genus and *Bdellovibrio* genus (Asaf et al., 2020). In addition, it has been reported that *Sphingomonas* have a variety of functions, including the potential to improve plant growth under environmental stress as well as to produce plant growth hormones (Asaf et al., 2020). Furthermore, due to its high tolerance to environmental stress, different species of *Sphingomonas* were isolated from various soil environments, including mountain soil, organic-polluted soil, garden soil, and tomato garden (Feng et al., 2019; Wang et al., 2019; Zhou et al., 2019; Zhu et al., 2019; Akter and Huq, 2020; Maeng et al., 2020; Akter et al., 2021). Therefore, *Sphingomonas* could adapt to different fertilizer strategies, and due to its potential to produce plant growth hormones, it could affect cucumber growth and the abundance of other bacteria.

Additionally, according to the functional analysis, it was revealed that different fertilizer strategies significantly affect the bacterial function of biofilm formation, pathogenic, and stress tolerance. Fertilizer has been demonstrated to influence biofilm formation and the effect degrees dependent on the fertilizer types and microbial communities. For instance, the external nitrogen source exerted an inhibitory effect on the biofilm formation of *Xanthomonas oryzae pv*. *Oryzae* (Ham and Kim, 2018). Tao et al. (2020) reported that the bio-organic fertilizer could accelerate the abundance of Pseudomonas and promote the formation of biofilm between specific bacterial taxa, which benefits the resistance to plant disease. All fertilizer strategies exerted significant promotion of the abundance of biofilm formation-related bacteria, where the 30%-reducing NPK with M showed the strongest effect. Besides, fertilizer strategies were also found to affect the bacterial types. The abundance of aerobic and Gram-negative bacteria was enhanced by fertilizers, while the anaerobic and Gram-positive bacteria were suppressed. Meanwhile, the optimized combined fertilizer strategy showed the strongest effects. In a previous study, it was reported that soil organic matter, especially the levels of soil carbon could strongly influence the abundance of aerobic and Gram-negative bacteria (Si et al., 2017). Herein, the DNPKM showed the highest SOM, which was responsible for the abundance of aerobic and Gram-negative bacteria.

For the composition of the fungal community, saprotrophs showed a high abundance in all treatments, and *Gymnoascus* was identified as the most abundant genus. Saprotrophs could decompose the residues of animals and plants and organic fertilizer and therefore improve the level of soil humus, which is consistent with the increasing SOM observed in the presence of different fertilizer strategies. Primarily, the isolation of *Gymnoascus* was found to suppress the growth of *Bacillus subtilis* and *Septoria nodorum* and inhibit the activity of *Haemonchus contortus* (Clark et al., 2005, 2006). The metabolites of *Gymnoascus* were reported to affect plant growth and the effect depending on its concentration. Therefore, the fertilizer strategies might influence the abundance and metabolism of *Gymnoascus* and further affect the composition and structure of the fungal community in the soil.

### Outlook

Soil properties play critical roles in the fluctuations of the microbial community composition, and it is considered to be the critical factor linking fertilizer strategies and microbial community. Therefore, the correlation between soil properties and microbial communities should explored elaborately in the upcoming studies, which can reveal the mechanism underlying the function of fertilizers. On the other hand, soil layers also play vital roles in the evolution of microbial communities. This study is focused on the response of topsoil to the fertilizer strategies, and it is also necessary to reveal the changes in the deep soil properties and microbial communities.

## Conclusion

Our results demonstrated that the DNPKM approach (30%-reducing chemical fertilizer combined with manure) substantially improve the yield and quality of cucumber as much as possible without damaging the soil health., even though the input of chemical fertilizers was reduced by nearly one third. The new approach improved the abundance of aerobic, biofilm formation-related, and Gram-negative bacteria and suppressed the anaerobic and Gram-positive bacteria. Moreover, compared with the traditional method, the new approach can improve the quality of cucumber and reduce the planting cost, which is very suitable for the current demand and has a widely promising application prospect. Because of this, we provide a new option for sustainable and green agricultural development.

## Data availability statement

The datasets presented in this study can be found in online repositories. The names of the repository/repositories and accession number(s) can be found in the article/Supplementary material.

## Author contributions

MW, LJ, and QT: conceptualization. MW, YX, HN, and NL: methodology. YX, SR, and YML: software. MW, YX, HN, YW, YY, and JS: validation. MW, YX, ZL, and YCL: formal analysis. MW, YX, LJ, and QT: investigation. YZ, LJ, and QT: resources. MW and HN: data curation. MW, YX, HN, and QT: writing—original draft preparation. YZ, LJ, and QT: writing—review and editing. YX and HN: visualization. LJ and QT: supervision. YZ, LJ, and QT: funding acquisition. All authors have read and agreed to the published version of the manuscript.

## Funding

This research was supported by funding from the National Key R&D Program of China (Grant No. 2021YFD1900900, 2019YFA0904000), Taishan Industrial Leading Talents Project (2020), Shandong- high efficiency ecological agriculture innovation project (LJNY202124), Basic long-term monitoring of agricultural microorganisms NAES-AM-025, the Recruitment Program of Global Experts (1000 Plan), Shandong Key Research and Development Program (2019JZZY010721, 2019JZZY010724), the Program of Introducing Talents of Discipline to Universities (B16030), Natural Science Foundation of Shandong Province (ZR2022MD081) and Innovation and Application of Key Technologies in Green, Simplified and High-quality Cultivation of Qudi Cucumber,” one of the top ten Agricultural Industrial Science and Technology Innovation Projects in Jinan.

## Conflict of interest

The authors declare that the research was conducted in the absence of any commercial or financial relationships that could be construed as a potential conflict of interest.

## Publisher’s note

All claims expressed in this article are solely those of the authors and do not necessarily represent those of their affiliated organizations, or those of the publisher, the editors and the reviewers. Any product that may be evaluated in this article, or claim that may be made by its manufacturer, is not guaranteed or endorsed by the publisher.

## Footnotes

^1^ http://www.arb-silva.de/

^2^ https://unite.ut.ee/
