# Supplementary material for: Effect of fertilization combination on cucumber quality and soil microbial community
Source: Front Microbiol. 2023 Feb 23;14:1122278. doi: 10.3389/fmicb.2023.1122278 (PMC9996052; doi:10.3389/fmicb.2023.1122278)
Supplement: Supplementary file 1 [file Data_Sheet_1.docx]

**Supplementary materials**

Effect of fertilization combination on cucumber quality and soil microbial community


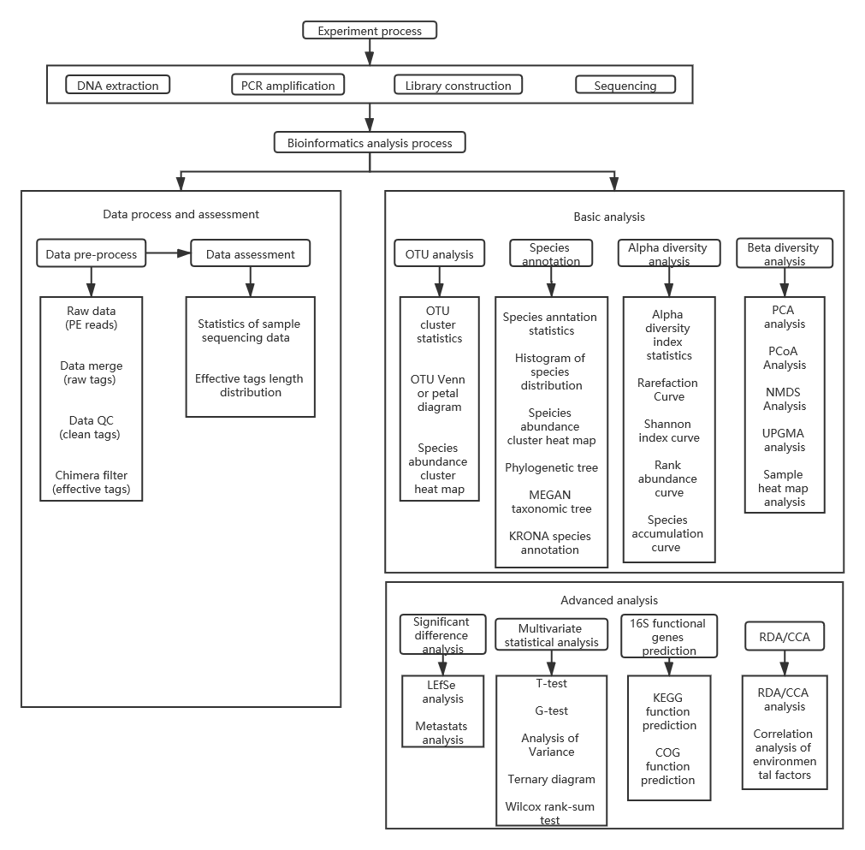


Figure S1. The process of sequence analysis.


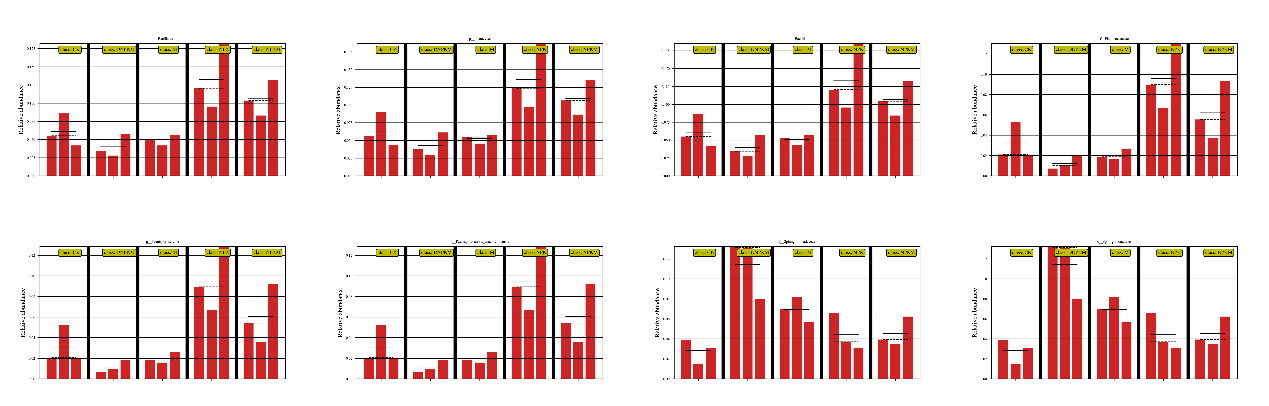


Figure S2. The specific abundance of the identified bacterial biomarkers in different treatments.


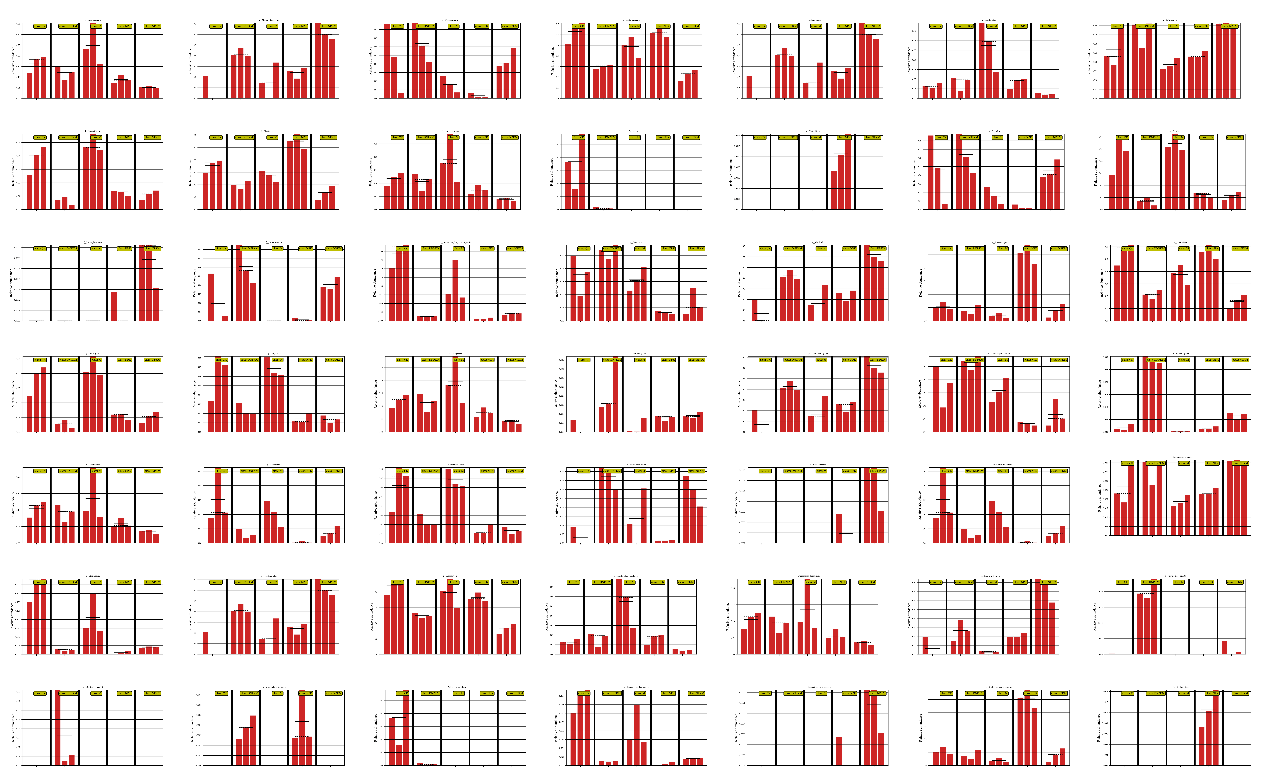


Figure S3. The specific abundance of the identified fungal biomarkers in different treatments.

|  | Planting | seeding | Variety |
| --- | --- | --- | --- |
| 1^st^ crop | September 17, 2018 | January 24, 2019 | Deruit Y2 |
| 2^nd^ crop | January 27, 2019 | June 10, 2019 | Jinyou 208 |
| 3^rd^ crop | September 15, 2019 | January 26, 2020 | Deruit Y2 |
| 4^th^ crop | January 28, 2020 | June 14, 2020 | Jinyou 208 |
| 5^th^ crop | September 17, 2020 | January 23, 2021 | Deruit Y2 |
| 6^th^ crop | January 25, 2021 | June 6, 2021 | Jinyou 208 |

Table S1. The planting and seeding time point of cucumber

| Layer  cm | NO_3_-N  mg/kg | NH_4_^+^-N  mg/kg | Total N  g/kg | Available P  mg/kg | Available K  mg/kg | Soil organic matter  g/kg |
| --- | --- | --- | --- | --- | --- | --- |
| 0-30 | 34.93 | 18.05 | 1.650 | 123.48 | 365.07 | 34.87 |

Table S2. Soil base physicochemical properties of experiment sites before treatment

Table S3. Sequences of used primer in PCR amplification

| Primers | Sequences |
| --- | --- |
| 338F | 5’-ACTCCTACGGGAGGCAGCAG-3’ |
| 806R | 5’-GGACTACHVGGGTWTCTAAT-3’ |
| ITS1F | 5’-CTTGGTCATTTAGAGGAAGTAA-3’ |
| ITS2R | 5’-GCTGCGTTCTTCATCGATGC-3’ |

Table S4. The cucumber production of each crop during the 3-year growth (ton/acre)

|  | 1^st^ | 2^nd^ | 3^rd^ | 4^th^ | 5^th^ | 6^th^ |
| --- | --- | --- | --- | --- | --- | --- |
| CK | 91.45 ± 4.98 | 150.50 ± 6.61 | 47.66 ± 1.65 | 130.21 ± 5.10 | 54.92 ± 3.04 | 131.34 ± 2.63 |
| M | 97.17 ± 4.40 | 152.22 ± 3.75 | 50.16 ± 3.07 | 135.34 ± 1.56 | 55.58 ± 1.08 | 134.88 ± 3.17 |
| NPK | 108.89 ± 0.49*# | 165.31 ± 4.32*# | 55.66 ± 3.23*# | 127.48 ± 3.70 | 70.75 ± 0.80*# | 140.73 ± 5.84 |
| NPKM | 106.45 ± 3.68*# | 161.63 ± 3.12*# | 51.88 ± 1.19 | 136.49 ± 2.16 | 70.47 ± 4.60*# | 133.17 ± 4.14 |
| DNPKM | 104.25 ± 4.86* | 154.00 ± 2.72 | 50.53 ± 3.22 | 143.12 ± 5.04*# | 69.50 ± 4.85*# | 134.84 ± 0.77 |
